# Formation of UHMWPE Nanofibers during Solid-State Deformation

**DOI:** 10.3390/nano12213825

**Published:** 2022-10-29

**Authors:** Ramin Hosseinnezhad, Iurii Vozniak, Dario Romano, Sanjay Rastogi, Gilles Regnier, Ewa Piorkowska, Andrzej Galeski

**Affiliations:** 1 Centre of Molecular and Macromolecular Studies, Polish Academy of Sciences, 90363 Lodz, Poland; 2 Faculty of Physical Sciences and Engineering, Division of Chemical Sciences, King Abdullah University of Science and Technology (KAUST), Thuwal 23955-6900, Saudi Arabia; 3 Procédés en Ingénierie Mécanique et Matériaux Laboratory, PIMM, Arts et Métiers, CNRS, CNAM, HESAM Université, 75013 Paris, France

**Keywords:** nanofibers, melting, composites, UHMWPE, crystals, disentangled

## Abstract

A network of nanofibers is formed in situ through solid-state deformation of disentangled ultra-high molecular weight polyethylene (dis-UHMWPE) during compounding with a polyolefin elastomer below the melting temperature of dis-UHMWPE crystals. Dis-UHMWPE was prepared in the form of powder particles larger than 50 μm by polymerization at low temperatures, which favored the crystallization and prevention of macromolecules from entangling. Shearing the blend for different durations and at different temperatures affects the extent to which the grains of dis-UHMWPE powder deform into nanofibers. Disentangled powder particles could deform into a network of nanofibers with diameters between 110 and 340 nm. The nanocomposite can be further sheared for a longer time to decrease the diameter of dis-UHMWPE nanofibers below 40 nm, being still composed of ≈70 wt.% of crystalline and ≈30 wt.% of amorphous components. Subsequently, these thinner fibers begin to melt and fragment because they are thinner and also because the amorphous defects locally decrease the nanofibers’ melting temperature, which results in their fragmentation and partial loss of nanofibers. These phenomena limit the thickness of dis-UHMWPE nanofibers, and this explains why prolonged or more intense shearing does not lead to thinner nanofibers of dis-UHMWPE when compounded in a polymeric matrix.

## 1. Introduction

All-polymer nanocomposites are still unique and usually prepared by dispersing ready-made polymer nanofibers or nanodroplets in a polymer matrix. There are several techniques for the production of polymer nanofibers or nanodroplets, for instance, hard template synthesis and soft template synthesis [1,2]. Hard template synthesis is the polymerization of micro-/nanotubes or wires within cavities of other materials. A post-synthesis process is needed for removing the templates. The method was applied to synthesize nanotubes/wires of a variety of conductive polymers such as polyaniline [3,4] and polypyrrole [5]. The soft template methods are based on self-assembly mechanisms due to hydrogen bonding, van der Waals forces, or electrostatic interactions as driving forces, e.g., [6].

Electrospinning is an effective approach to fabricating long polymer fibers with diameters from micrometers down to even a few nanometers by taking advantage of strong electrostatic forces [7]. This process is particularly suited for the production of fibers of large and complex molecules, especially since it allows the production of continuous long nanofibers. Electrospinning from molten polymers is also practiced. There are just a few reports on the electrospinning of some types of polyethylenes [8,9,10], but none on UHMWPE. Nanofibers with good molecular orientation have been fabricated [11,12], and such polymer nanofibers have been used to produce polymer nanocomposites and enhance their mechanical properties, for instance [13,14]. Fakirov et al. [15] developed a three-step method to fabricate a nanocomposite structure. The procedure comprises: (1) melt blending of two immiscible polymers; (2) spinning of the blend into fibers; and (3) remelting the matrix without melting of fibrous inclusions to obtain an all-polymer composite with fibrous reinforcement.

Voznyak et al. [16] developed a method of fabricating all-polymer nanocomposites by shearing an immiscible polymer blend at such processing conditions that dispersed polymer inclusions undergo strong shear deformation with simultaneous shear-induced crystallization. The method was illustrated in the case of polylactide with dispersed polybutylene succinate (PBS) inclusions that were sheared and longitudinally deformed in the extruder with simultaneous cooling in a slot capillary, and PBS nanofibers were formed and crystallized. Shearing of PBS increases the nonisothermal crystallization temperature by as much as 30 °C. Hosseinnezhad and Voznyak obtained several polylactide-based nanocomposites according to that procedure: with aliphatic–aromatic co-polyesters [17], polyhydroxyalkanoate [18], and polyamides [19,20] succeeding in the formation of nanofibers by strong shearing of a blend of the two immiscible polymers and followed by shear-induced crystallization of nanofibrillar inclusions.

Another method for fabrication of fibrillar nanocomposites, developed several years ago, comprises an addition of a crystalline polymer in a powder or flake form to another thermoplastic polymer prior to its processing, with the thermoplastic polymer selected from a group consisting of polyolefins, polyacetals, vinyl polymers, polyamides, or polyesters [21,22,23]. The other polymer should be highly crystalline with low macromolecular chain entanglement in the amorphous phase. Such requirements are fulfilled by PTFE reactor powder constituting very large chain-extended crystals having a melting temperature close to the equilibrium melting temperature. PTFE fibrils can nucleate the crystallization of a matrix polymer and influence the mechanical properties of the nanocomposite [21,22,23,24,25]. The obtained nanocomposite can be processed further by extrusion, injection, thermoforming, or foaming in order to produce useful items. Previous attempts at the formation of polymer nanocomposites with fibrillar inclusions by compounding were unsuccessful because it was impossible to deform solidified crystalline polymer inclusions during compounding. In another study, Krajenta et al. [26] generated an all-polymer nanocomposite using disentangled polypropylene in the form of grains. Polypropylene with disentangled macromolecules was obtained by rapid crystallization from dilute solutions. It was possible to initiate its plastic flow at low shear stresses when dispersed in molten polystyrene. The grains of disentangled polypropylene were deformed to long nanofibers or rather nano-tapes. Sheared crystals become deformed and retain their shape when the shear is decreased. The higher the capillary number, *Ca*, the ratio of viscous drag forces versus surface tension forces acting across an interface, the more efficient the stress transfer from sheared polymer matrix to inclusions.

*Ca* = *η* × *U*/σ (1)

where *η* is the viscosity, *U* is the characteristic velocity of the material, and σ is the interfacial tension.

In order to deform polymer crystals embedded in a viscous medium, critical shear stress, resolved in a slip plane of the respective crystal slip systems, must be reached and exceeded. In the past, the possibility of deformation of a crystal embedded in a viscous matrix under shearing flow was assessed [27]. Shearing of the matrix was found to lead to mechanical instability of a crystal’s first layer which results in a continuous decrease in the coherence length of crystalline order in the sliding layers as the shear increases. The shear modulus and shear yield stress of the sheet crystal was at the level of 2 MPa, a value characteristic for crystals of many polymers at temperatures slightly below their melting temperatures. See also the yield stresses for polyethylene crystals measured as functions of the temperature and the deformation rate [28,29,30]. The above estimations make the idea of transforming polymer crystals into nanofibers by shear deformation quite realistic. It has been clearly established that polyethylene crystals undergo the (100)<001> chain slip initiated by dislocations at the edges of crystals [31]. This is the easiest slip since the other, (100)<100> transverse slip requires significantly larger shear stress e.g., [29,32,33,34,35]. Hence, polyethylene chains align in the shearing direction due to such slip along chains and form fibers. In 2007 we discovered that PTFE reactor-grade powder is deformed into fibers during blending [36], and later by blending with other polymeric matrices we arrived at the new “all-polymer” nanocomposites [21,22]. The structure of PE crystals is very similar to PTFE crystals in the sense that the easiest crystallographic slips are slips in the chain direction leading to easy fibrillation.

The stress in a molten polymer matrix is simple, being nearly pure shear stress. The shear stress component resolved in (100) planes of polyethylene crystals can cause crystallographic chain slip (less efficient transverse slip) if its value in the chain direction is sufficient. No other slips are allowed because slip planes cannot cross macromolecular chains. At room temperature, the critical shear stress value for PE (100)<001> chain slip is around 7 MPa. However, crystallographic slips are thermally activated, and at compounding temperature, their level is only 1–2 MPa.

However, for fibrous shape, the deformation of polymer crystals to large strains is needed. This is possible when the density of entanglements persisting in the surrounding amorphous phase is drastically reduced. One of the means is polymerization at a lower temperature to allow crystallization during polymerization [37,38]. The aim of this study is to find a way to strongly deform polyethylene inclusions during compounding and preserve their expected fibrous shape, i.e., to act against capillary instabilities. A range of trials with regular commercial polyethylene was performed but there are limits of 2 to 4 of achievable deformation and no fibrous shape was reached. Hence, a further selection of polymer powder grains was based on the criteria of low-chain entanglements along with their high crystallinity. This state can be achieved for polyethylene by crystallization under pressure e.g., [39], crystallization from solutions (e.g., DSM technology for Dyneema ultra strong PE fibers), and crystallization during polymerization based on single-site catalyst [40,41]. Polymerized PE powder grains can possess either chain-folded or chain-extended crystals and can be drawn to ultra-high strains if the polymer chains are unentangled. If the number of catalytically active sites is very low and/or the polymerization temperature is far below the melting temperature, the polymerization rate becomes lower than the crystallization rate, and it is possible to reach the state when growing polymer chains can be considered as separated from each other. This results in an independent growth of monomolecular crystals—a single chain forming a single crystal [42]. Polymer crystals grown during polymerization are called nascent or polymerized crystals [43]. Because of the reduced density of entanglements and chain-extended fashion, easy deformation of PE by moderate shearing is possible. However, materials with chain-folded crystals, even with unentangled macromolecules when melted and recrystallized, lose entirely the advantageous ultradrawing feature. This confusing phenomenon was considered by Barham and Sadler [44] in the studies of the melting of chain-folded crystals. Using neutron scattering and deuterated PEs they measured the changes in the radius of gyration. For chain-folded crystals, the radius of gyration is rather low and upon melting it suddenly inflates to the equilibrium value of a random coil. The expansion of chains is very rapid, less than a few seconds, and pays no attention to the neighboring chains, which is in contrast to the reptation theory based on the reptation tube formed by fragments of neighboring chains. The phenomenon rapidly leads to a significant increase in the number of entanglements and the loss of high drawability.

The above introduction is aimed at explaining the possibility of effective deformation of UHMWPE crystals by shearing, especially in the extruder. That was estimated based on the knowledge of plastic deformation of PE crystals and on the shear field strength. The drag forces exerted on the polymer powder grains were compared with the shear stresses needed to deform polyethylene crystals at a higher temperature. The constraints from the surrounding amorphous PE material were drastically reduced due to disentangling of macromolecules. The transformation of powder grains into fiber-shaped entities was predicted due to the easiest crystallographic slips in the chain direction due to PE crystal plasticity. Finally, the possibility of forming a network of nanofibers through solid-state deformation of nascent powder of ultra-high molecular weight polyethylene (UHMWPE), with disentangled macromolecules, is suggested.

In the present work, the possibility of forming a network of nanofibers through solid-state deformation of nascent powder of ultra-high molecular weight polyethylene (UHMWPE), with disentangled macromolecules, is explored. The nanofibers are formed in situ during compounding with a polyolefin elastomer at a temperature below that of the melting of the nascent UHMWPE. The influence of shearing time and temperature on the resulting nanofibers is studied. The decrease in fiber diameters and crystal sizes results in a decrease in the melting temperature of the crystals and is interpreted according to the modified version of the Gibbs–Thomson equation.

## 2. Materials and Methods

A commercial grade of ethylene–octene copolymer (supplied by The Dow Chemical Company, Gales Ferry, CT, USA) with the formula (CH_2_CH_2_)*x* [CH_2_CH[(CH_2_)_5_CH_3_]] *y* was used as a polymer matrix. This polyolefin elastomer (EOC) with the trade name Engage 8842 is an ultra-low-density copolymer combining exceptional properties of an ultra-low-density elastomer with the potential possibility of handling this polymer in pellet form. This EOC, with a density of 0.857 g cm^−3^, octene content of 45 wt.%, a melting temperature of 44 °C, and a melt flow index of 1 g/10 min (190 °C/2.16 kg, ASTM D1238) was chosen to deform the powders of disentangled UHMWPE to nanofibers in the solid state. Disentangled ultra-high molecular weight polyethylene (dis-UHMWPE), with a molecular weight of 1.4 million g mol^−1^, melting peak temperature (T_m_) of 140 °C, and melting enthalpy of 200 J g^−1^ (≈70 wt.% crystallinity), was used as synthesized and described by us earlier [41].

Composites of EOC/UHMWPE (95/5 wt.%) were prepared in situ by compounding EOC with dis-UHMWPE powder at a temperature higher than the melting temperature of the EOC matrix but lower than the melting temperature of the UHMWPE crystals to allow the formation of UHMWPE nanofibers in the solid state. The 5 wt.% dis-UHMWPE concentration was chosen to ensure the formation of a continuous network structure of nanofibers [20] and to minimize agglomeration of generated nanofibers during compounding. To reduce the agglomeration of UHMWPE particles, a masterbatch of EOC pellets ground at low temperature with 20 wt.% of UHMWPE was prepared by compounding in a co-rotating twin-screw extruder 2 × 20/40D EHP (Zamak Mercator, Skawina, Poland) operating at 20 rpm. The temperature zones in the extruder barrel were set at 60 °C. In order to obtain 5 wt.% of UHMWPE nanofibers in the final material, the EOC/UHMWPE masterbatch was diluted with EOC by compounding using the same corotating twin-screw extruder operating at 30 rpm. Two temperatures of 75 °C and 115 °C were set in the extruder barrel. UHMWPE nanofibers were formed during both preparation of the masterbatch and subsequent diluting by shearing extrusion. It is estimated, based on the screw cylinder clearance and screw rotation speed, that the material during diluting the masterbatch was subjected to shear at cylinder walls with the mean rate of 350 s^−1^, while between screws the shear rate exceeded 700 s^−1^.

The morphology of in situ generated composites was investigated with a JSM-5500 LV scanning electron microscope (SEM, Jeol, Tokyo, Japan)). The internal structure of the samples was exposed after a fracture in liquid nitrogen. The cryo-fracture surfaces of samples were coated with 20 nm thick gold layers by ion sputtering (JFC-1200, Jeol, Tokyo, Japan) and examined with SEM in a high vacuum mode at the accelerating voltage of 10–20 kV.

The melting and non-isothermal crystallization of the disentangled UHMWPE powder, EOC, and EOC/UHMWPE composites were examined by employing an indium-calibrated differential scanning calorimeter DSC Q20 (TA Instruments, New Castle, DE, USA). Samples weighing 6–10 mg were heated to 180 °C, annealed for 3 min, and cooled down to crystallize EOC, at the constant heating/cooling rate of 10 °C min^−1^. The entire thermal treatment was performed under nitrogen flow. The melting temperature (*T_m_*), the onset temperature of crystallization (*T_o_*), the endset temperature of crystallization (*T_e_*), and melting enthalpy (Δ*h_m_*) were determined based on the thermograms. The crystallinity (*X_c_*) of UHMWPE in the composites of EOC/UHMWPE was calculated as [45]:

*X_c_* (%) = [Δ*h_m_*/(*Φ* × Δ*h_m_*^0^)] × 100% (2)

where, *Φ* is the weight fraction of UHMWPE in the EOC/UHMWPE composites and Δ*h_m_*^0^ is the heat of fusion of a 100% crystalline UHMWPE taken as 293 J g^−1^ [46].

## 3. Results

### 3.1. Morphology of Nanocomposites

**Figure 1:**
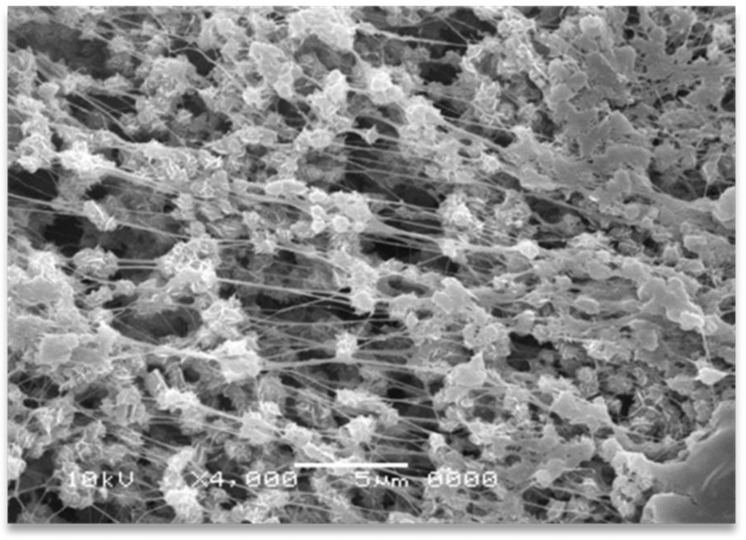
SEM micrographs of dis-UHMWPE powder grains sintered at 30 °C under 60 atm (without any melting) and subjected to slight tensile deformation during removal from the mold.

The effect of shearing time and temperature on the in situ solid-state formation of UHMWPE nanofibers was scrutinized by compounding the solid dis-UHMWPE powders with the molten EOC in a mini twin-screw extruder. The low *T_m_* of the employed EOC is a specific feature facilitating the solid-state deformation of dis-UHMWPE into nanofibers by simple compounding. The easy deformation ability of dis-UHMWPE is illustrated in Figure 1, where the SEM image of a sintered dis-UHMWPE powder is subjected to accidental tensile deformation while being removed from the mold. Many tiny, sub-micron-sized fibers were spun from dis-UHMWPE grain particles. The sintering was performed at 30 °C and 60 atm, and it was far from melting.

**Figure 2:**
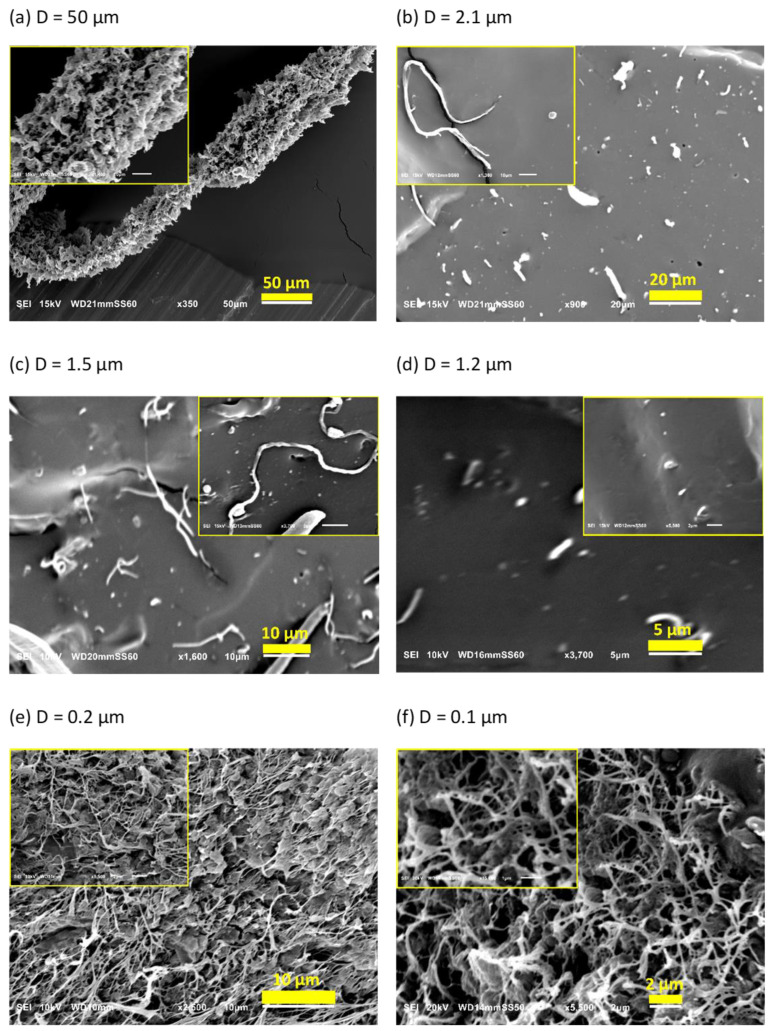
SEM images of: (**a**) dis-UHMWPE powder, and EOC/UHMWPE (95/5) composites processed with the shear rate of 350 s^−1^: (**b**) at 75 °C for 10 min, (**c**) at 75 °C for 30 min, (**d**) at 75 °C for 90 min, (**e**) at 115 °C for 10 min, and (**f**) at 115 °C for 30 min.

The morphology of dis-UHMWPE powder is presented in Figure 2a. The anisotropic particles, with an average diameter (D) of 50 μm, constitute the vast fraction of dis-UHMWPE powder. The image with higher magnification, in the inset of Figure 2a, reveals that the particles are agglomerates of sub-grains, being less than 5 μm in diameter. Apparently, each grain consists of several large lamellar particles, loosely stacked, and, as already shown, capable of single fiber drawing. This morphology may result from the synthesis conditions, i.e., polymerization, using a specific post-metallocene catalyst [46]. The polymerization conditions were set such that the crystallization occurs and hinders the entanglement of chains. The presence of less entangled non-crystalline regions in the semi-crystalline polymer increases the probability of the formation of fibrous morphology. The deformation of dis-UHMWPE grains into nanofibers should be primarily caused by the viscous drag from sheared EOC matrix. As mentioned in the Introduction, the best parameter describing the action of viscous drag against interfacial forces and further against shear plastic resistance of UHMWPE crystals is the capillary number (see Equation (1)). The complex viscosities of the EOC matrix, that was sheared in the extruder at 350 s^−1^, were equal to 1693 Pa·s and 1044 Pa·s, for 75 °C and 115 °C, respectively. The viscosities of EOC were measured using a strain-controlled rheometer, ARES LS2, TA Instruments, for shear rate of 350 s^−1^. The characteristic velocity of the material in the extruder was at the perimeter of the extruder screws which is equal to the screw circumference times its rotation rate, 2 × π ×10 mm ×30 rev min^−1^ = 31.42 mm s^−1^. Assuming the interfacial tension at the level of 11.8 mN m^−1^ for polyethylene crystal lateral face [47,48], the capillary numbers are 4500 and 2780 for 75 °C and 115 °C, respectively. The values of the capillary numbers are large, indicating that viscous drag exceeds multifold times the interfacial tension of polyethylene crystals, in turn indicating the predominance of viscous drag forces over interfacial forces. The shear stress acting on dis-UHMWPE grains can be estimated from acting shear rates and the viscosity of the EOC matrix: it is around 2 MPa during extruder processing at 75 °C and around 1 MPa at 115 °C. The yield tress is a direct measure of plastic resistance of polymer crystals. Several reports [34,49] have disclosed the yield stress of polyethylene crystals at around 3 MPa at 75 °C and at around 1 MPa at 115 °C. Those stresses are tensile in character, while the yield is caused by crystal shear at ±45° caused by the shear stress being one half of the tensile stress. The shear stresses required for polyethylene crystal deformation are of similar values as the respective shear stresses acting in the extruder, even lower than the one generated in the zone between extruder corotating screws. Hence, the plastic resistance of polyethylene crystals can be overpowered, and dis-UHMWPE grains can be strongly deformed during extrusion to form long fibers and nanofibers.

Figure 2b–f present SEM images of the cryo-fracture surface of EOC/dis-UHMWPE composites processed at 75 or 115 °C for 10, 30, or 90 min with the shear rate of 350 s^−1^. Figure 2b shows elongated inclusions, which evidences that the applied shear rate was high enough to achieve effective stress transfer from the molten matrix to the dispersed solid particles. The applied shear stress exceeded the critical value required for crystallographic slip and led to plastic deformation, which is favored by the low entangled state in the non-crystalline regions and the associated adjacent re-entry of chains [50]. Due to the high crystallinity and low chain entanglements in the non-crystalline regions, dis-UHMWPE powders were easily transformed into long fibers with an average diameter of 2.1 µm. This observation is in accordance with the ease of solid-state processing of these polymers [51]. However, as expected, the conditions during the preparation of the masterbatch affect the deformation ratio of nanofibers. Figure 2c,d reveal that solid-state deformation of dis-UHMWPE powders in molten EOC matrix depends on the shearing time. In particular, the longer the shearing time, the thinner the fibers obtained. It is observed in the images with higher magnification, in the insets of Figure 2b,c that increasing the shearing time from 10 to 30 min increases the fiber aspect ratio from 70 to 105.

An increase in the temperature from 75 to 115 °C results in the formation of much thinner fibrils with diameters varying from 110 to 340 nm. It should be noted that in this case, a physically entangled nanofibrous network is formed (Figure 2e). This is because with the decrease in nanofibril diameters and the increase in their length the probability of the formation of physical links between them increases. Figure 2f shows SEM images of the cryo-fracture surface of the EOC/dis-UHMWPE nanocomposite processed for 30 min at 115 °C. It is revealed that thicker nanofibers of Figure 2e, with an average diameter of 200 nm, split out from bundles and deformed further to a diameter ranging from 40 nm to 160 nm. Since the nanofibers were generated in situ in the solid state, the capillary instabilities and break-up into droplets did not occur. However, as it is further demonstrated, the particles were fibrillated to the extent that, despite the high *T_m_* of the nascent powder, the thus obtained fibers could melt at a decreased temperature.

**Figure 3:**
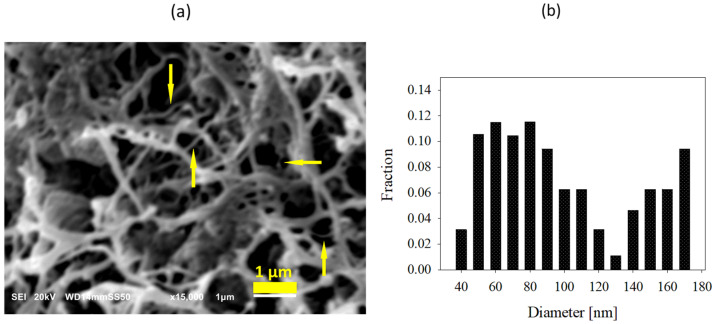
(**a**) SEM image of dis-UHMWPE nanofiber network in nanocomposite processed at 115 °C for 30 min. Thicker fibers are bundles consisting of several thinner nanofibers, as pointed out by arrows; (**b**) diameter distribution of the nanofibers.

Figure 3 shows the SEM micrograph and diameter distribution of nanofibers obtained via the deformation of dis-UHMWPE particles by shearing during mixing with molten EOC at 115 °C for 30 min. It is seen that dis-UHMWPE nanofibers with a diameter ranging from 40 nm to 170 nm were obtained. The figure shows that the thicker fibers might be bundles consisting of several, usually more than two, thinner nanofibers.

The deformation of dis-UHMWPE grains into nanofibers was primarily caused by the viscous drag from the sheared EOC matrix. As mentioned in the Introduction, the best parameter describing the action of viscous drag against interfacial forces and further against shear plastic resistance of dis-UHMWPE crystals is the capillary number. The complex viscosities of the EOC matrix, that was sheared in the extruder at 350 s^−1^, were equal to 1693 Pa·s and 1044 Pa·s, for 75 °C and 115 °C and for the shear rate of 350 s^−1^, respectively. (Viscosities were measured using a strain-controlled rheometer, ARES LS2, TA Instruments.) The characteristic velocity of the material in the extruder was the perimeter of the extruder screw times its rotation rate, 30 rev min^−1^ × 2 × π × 10 mm = 31.42 mm s^−1^. Assuming the interfacial tension at the level of 11.8 mN·m^−1^ for polyethylene crystal lateral face [47,48] the capillary numbers are 4500 and 2780 for 75 °C and 115 °C, respectively. The values of the capillary numbers are large, indicating that viscous drag exceeds the interfacial tension multifold times.

### 3.2. Melting of Nanofibers

The melting of polymer crystals is usually described by the Gibbs–Thomson equation. The in situ generated nanofibers are characterized by a large surface area, which can influence their melting behavior. It seems reasonable to assume that UHMWPE crystals in the highly deformed nanofibers are aligned with their c-axes parallel to the fiber axes. Hence, the melting process is influenced not only by the interfacial free energy of lamellae basal planes in contact with the amorphous phase, σ_e_, but also by the interfacial free energy of (hk0) planes in contact with the surrounding polymer melt, σ_sl_,. Both of these components influence the Gibbs free energy (Δ*G*) of the crystalline phase of the fibers:

Δ*G* = *πDLσ_sl_* + *2π(D/2)^2^ σ_e_* − *π(D/2)^2^L* Δ*g_f_* (3)

where Δ*g_f_* is the bulk free enthalpy of the volume unit of a nanofiber without surface effects, *D* is the diameter of nanofiber, and *L* is the crystal thickness. Melting occurs when Δ*G* = 0. Taking into account that Δ*g_f_* = Δ*s_f_* Δ*T* = Δ*h_f_*^0^ Δ*T*/*T_m_*^0^, where Δ*h_f_*^0^ is the heat of fusion per unit volume, one obtains:

Δ*T* = *T_m_*^0^ − *T_m_* = Δ*T*_1_ + Δ*T*_2_ (4)

and:

Δ*T*_1_= 2*T_m_*^0^
*σ_e_*/(*L* Δ*h_f_*^0^) (5)

Δ*T*_2_= 4*T_m_*^0^*σ_sl_*/(*D* Δ*h_f_*^0^) (6)

For lamellar crystals *D* >> *L* Δ*T*_2_ = 0, hence Equation (4) takes the form of the Gibbs–Thomson equation, whereas for *L* >> *D* Δ*T*_1_ = 0, and the modified version of the Gibbs–Thomson equation is obtained [52].

**Figure 4:**
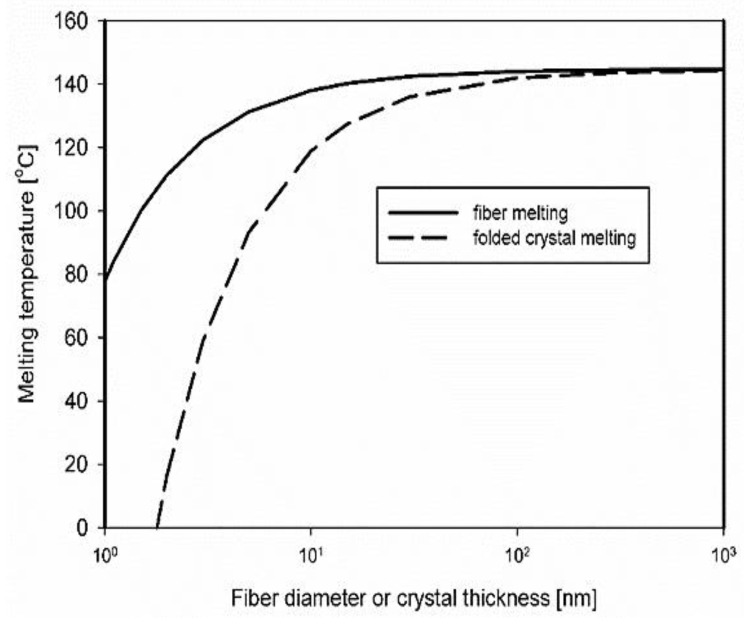
Depression of melting temperature due to the contribution of interfacial free energies of lateral surfaces of dis-UHMWPE crystals in the nanofibers embedded in EOC and due to surfaces consisting of chain folds. (Thermal data for polyethylene: *σ_sl_* = 11.8 mN/m, *σ_e_* = 90 mN m^−1^, *T_m_*^0^ = 144.5 °C, and Δ*h_f_*^0^ = 293 J cm^−3^ taken from [46,47,53]).

Considering that EOC has a chemical structure very close to that of polyethylene, we can assume that the crystal surface free energy in molten EOC is very close to that in polyethylene in the molten state. Moreover, it is reasonable to assume that the polymer chain axes of dis-UHMWPE nanofiber, which was formed by the plastic deformation of chain-extended crystals, are parallel to the fiber axis. Thus, the interfacial tension between the cylindrical lateral face of dis-UHMWPE nanofiber and the EOC matrix, *σ_sl_*, can be considered similar to that between the lateral face of crystal and the rubbery amorphous phase of polyethylene, which was assessed to be 11.8 mN m^−1^ [47,48]. Figure 4 depicts the depression of *T_m_* due to interfacial free energy of dis-UHMWPE crystals in the form of nanofibers embedded in EOC, according to Equation (6), that is when the effect of the basal planes of the crystals is not taken into account. For the crystals with finite thickness, the additional decrease in *T_m_* should occur by Δ*T*_2_ expressed by Equation (5), according to the Gibbs–Thomson equation. It is evident that the *T_m_* of crystalline nanofibers with a diameter in the range of a few tens of nanometers is significantly reduced, and could occur during compounding, even at a temperature below *T_m_* of the nascent dis-UHMWPE. 

A very strong depression of melting temperature for high interfacial free energy indicates that the defects on the nanofiber surfaces, such as chain folds, amorphous fragments, etc., could decrease the melting temperature locally, leading to local nanofiber melting and fragmentation. It must be remembered that the total crystallinity of dis-UHMWPE is at the level of 70 wt.%, and 30% of amorphous fragments are then dispersed along the nanofibers, also decreasing the crystal thickness of a nanofiber.

### 3.3. Melting Behavior of Nanocomposites

**Figure 5:**
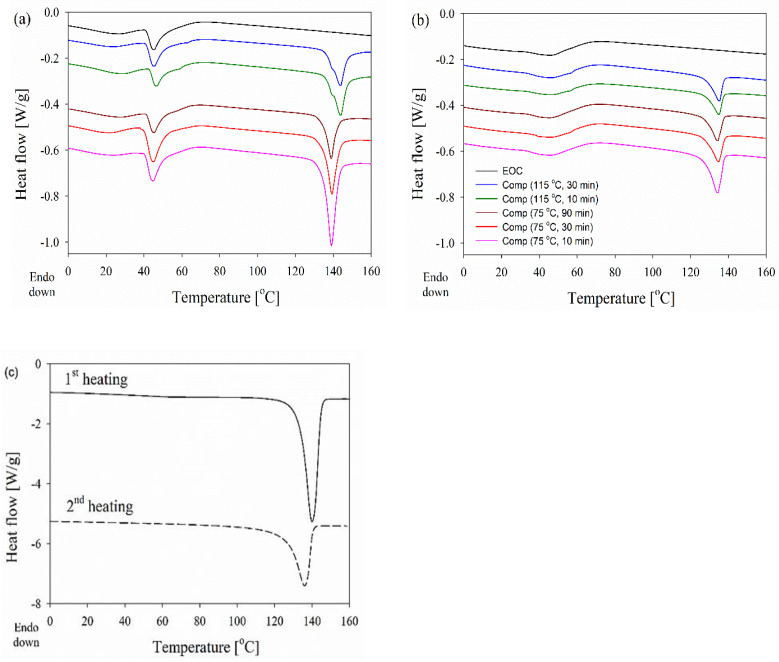
DSC curves obtained at 10 °C min^−1^ during the first (**a**) and second (**b**) heating of EOC, and EOC/UHMWPE nanocomposites. The melting endotherm of dis-UHMWPE is separately presented (**c**) for clarity.

The first heating and the second heating thermograms, measured at 10 °C min^−1^, of dis-UHMWPE powder, EOC, and EOC/UHMWPE composites are shown in Figure 5.

Table 1 compares T_m_, melting onset (*T_o_*), and endset (*T_e_*) temperatures and the degree of crystallinity (*X_c_*) calculated based on the first and second heating thermograms.

The *T_m_* of UHMWPE powder recorded during the first heating was at 140 °C and decreased to 136 °C during the second heating, which corresponds to the crystal thickness, calculated based on the Gibbs–Thomson Equation (5) (with σ_e_ of 90 mN m^−1^ [48]) of 57 nm and 30 nm, respectively. In parallel, the crystallinity degree decreased from 68% to 45%.

**Figure 6:**
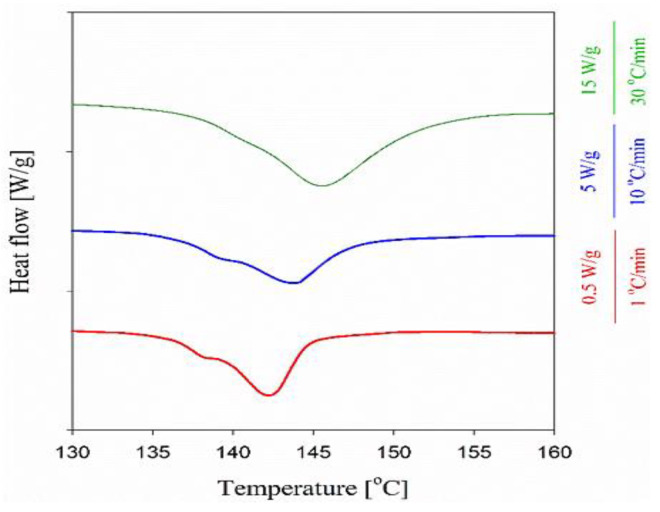
First heating thermograms (endo-down) of the nanocomposite, processed at 115 °C for 30 min, recorded at different heating rates.

All the composites, processed for different durations and at different temperatures well below *T_m_* of the dis-UHMWPE powder, clearly showed two distinct melting regions, i.e., below 70 °C and from 128 °C to 150 °C. The first region with a distinct peak at 46 °C is attributed to the melting of EOC crystals and remains almost unchanged independently of processing conditions, and is visible on the thermogram of neat EOC. During the second heating, the melting endotherm of EOC was different but still visible on all thermograms in the low-temperature range. The main effect of composite processing conditions and the resulting structure is on the melting of dis-UHMWPE crystals, showing different values of *T_m_* and Δ*h_m_*. The melting peaks of nanofibers in the range of 100–200 nm, in the nanocomposites, which had been processed at 115 °C, with *T_m_* of 144 °C, were featured with shoulders at ca. 140 °C. *T_o_* and *T_e_* were near 136 °C and 149–150 °C, respectively. *T_m_* of 144 °C corresponds to the lamellar thickness of 513 nm, according to Equation (5). Figure 5 compares the first heating thermograms of the nanocomposite processed at 115 °C for 30 min recorded at different heating rates. It appears that the *T_m_* increased whereas the low-temperature shoulder decreased with increasing heating rate, and only a trace of the latter remained during heating at 30 °C min^−1^. The increase in *T_m_* evidences that its high value did not result from annealing during heating, as faster heating limits reorganization processes in the crystalline phase. Although the high *T_m_* could partially result from the effect of constraints on the melting process, it undoubtedly shows that the crystal thickening and perfection occurred in dis-UHMWPE crystalline nanofibers during processing upon shearing at 115 °C. The crystal thickening should be accompanied by an increase in *X_c_*. However, after 10 min processing at 115 °C, *X_c_* was below that of dis-UHMWPE powder, 38%, and decreased to 34% after an additional 20 min of shearing. This evidences that in parallel to the thickening and perfection of dis-UHMWPE crystals another process could occur. Figure 6 compares well the thermograms of composites with dis-UHMWPE with the same thermal history, but annealing at 75 °C and 115 °C, and proves that annealing alone (without shearing) does not significantly affect later melting.

We hypothesize that the thinnest fibers formed upon shearing were unstable, melted during the processing, and crystallized during subsequent cooling with lower *X_c_*. Figure 4 shows that the fibers with diameters of a few nanometers could melt at *T_m_* lower by ca. 20 °C than the thick fibers containing crystals of the same thickness. Considering that on heating at 10 °C min^−1^ the melting of the nascent UHMWPE powder began close to 125 °C, and that temperature during processing could increase due to shearing, the melting of the thinnest fibers during processing seems to be highly probable.

In turn, the thermogram of the composite processed at 75 °C for 10 min is characterized by a single sharp melting peak with the *T_m_* of 139 °C, which mainly corresponds to the melting of polyethylene crystals with an approximate thickness of 30 nm, as estimated by the Gibbs–Thomson Equation (5), and Δ*h_m_* corresponding to *X_c_* of 62%. Shearing the composite at the same temperature but for longer times of 30 and 90 min changed neither the dis-UHMWPE melting peak temperature range nor *T_m_*, but *X_c_* decreased to ca. 40%.

The decrease in crystallinity could be also due to the formation of very thin fibers, which melt during processing. However, due to the low processing temperature, the decrease in crystallinity was rather related to the transformation process of UHMWPE powder into fibers. Unlike in the case of processing at 115 °C, the process was not accompanied by the thickening of crystals, which is reflected in the melting behavior similar to that of dis-UHMWPE powder. We note that others [54] have observed a decrease in crystallinity and melting temperature of poly(tetrafluoroethylene) (PTFE) during the shear-flow-induced transformation of PTFE powder into fibrils in a polypropylene matrix.

It is shown in Figure 5b that after melting and crystallization of the nanocomposite, the differences in melting of UHMWPE in the nanocomposite are lost, just by keeping the melt for a few minutes at 180 °C. Moreover, during the second heating of the composites, *T_m_* is 5–9 °C lower than *T_m_* during the first run. Figure 6 shows that regardless of the heating rate from 1 to 30 °C min^−1^ during the first heating of the EOC/UHMWPE composite, the melting peak is featured with a low-temperature shoulder. This evidences that the shoulder is related to the melting of a fraction of UHMWPE fibers rather than to recrystallization phenomena in the crystalline phase.

### 3.4. The Mechanism of Melting

**Figure 7:**
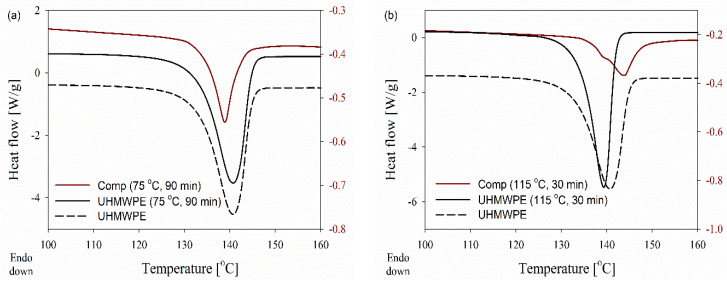
DSC curves obtained during the first heating of the disentangled UHMWPE and (95/5 wt.%) composite at 10 °C min^−1^, where dis-UHMWPE and the composite were annealed and processed respectively at: (**a**) 75 °C for 90 min, (**b**) 115 °C for 30 min.

**Figure 8:**
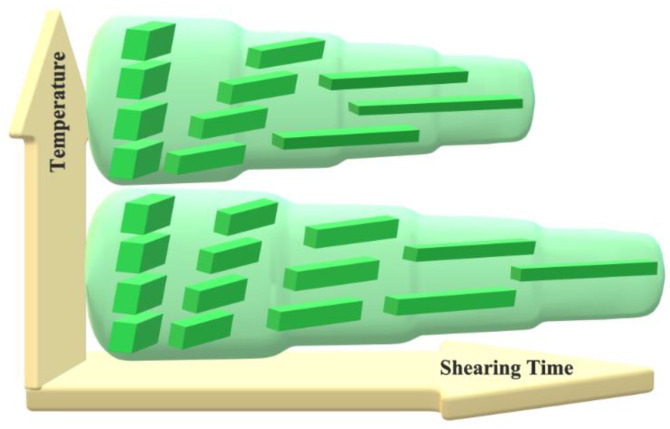
Schematic representation of the stages of dis-UHMWPE crystals during solid-state deformation into nanofibers. The crystals undergo crystallographic slip in the direction of macromolecular chains. The resulting fibers become thinner as the shearing time increases and they melt when they become too thin.

The disentangled UHMWPE obtained directly from a polymerization reactor is characterized by a low density of entanglements in the amorphous phase, which allows easy solid-state deformation. As a result, the powders undergo plastic deformation in a solid state and deform into micro and nanofibers. However, the initial morphology of crystals seems to be influenced during this deformation, which results in different thermal behavior upon melting. The melting of deformed disentangled UHMWPE powders in the range of 112 to 148 °C (see Figure 7a,b) assures the existence of unique chain-folded crystals with a thickness varying from 2 to 150 nm. The thin crystals in the range of a few nanometers, which are more vulnerable to the applied shear, break down and melt partially in the course of compounding the powder with EOC annealing. As it is suggested in Figure 8 for the composite processed at 75 °C for 10 min, the irregular stackings of lamellae in the initial powder are easily oriented and transformed into well-stacked lamellar crystals. This is facilitated by the lack of chain entanglements in the interlamellar amorphous phase. Shearing the composites for 30 and 90 min is accompanied by the formation of thinner fibers. However, the constant range and peak of melting from DSC prove that crystal thickness is undisturbed; on the other hand, the decrease in the enthalpy hints at the partial detachment of crystals and their subsequent integration within the amorphous region.

In the case of solid-state deformation at a higher temperature of 115 °C, stacked lamellar crystals ease both the local mobility and the diffusion of UHMWPE chains between neighboring crystals. This crystal thickening leads to the formation of thicker crystals and a shift in the melting of the new crystals to higher temperatures. Once the amorphous chain ties become elongated, the folded chains of thick crystals may be tilted to form chains of extended crystals. The formation of a double melting peak in DSC results reveals that the population of chain-extended crystals, attributed to the higher melting peak, is remarkable compared to the initial chain-folded crystals, i.e., shoulder peak. The existing chain-folded crystals still pose a melting peak at 138 °C while chain-extended crystals melt at a higher temperature of 144 °C due to the thickened lamellae (compare DSC melting endotherms for composites in Figure 7a,b). The melting of chain-folded crystals, which have a lower melting temperature on compounding at higher process temperatures and times, can be anticipated.

## 4. Conclusions

It appeared that the viscous drag forces subjected to the EOC matrix are significantly large to overcome interfacial forces and to develop sufficient shear stress at dis-UHMWPE grains to deform dispersed UHMWPE disentangled grains into long fibers and nanofibers. In fact, the capillary numbers for the two temperatures of processing, 75 and 115 °C, indicate that viscous drag forces exceed by several thousand times the interfacial tension, in turn indicating the predominance of viscous drag forces over interfacial forces and efficient transfer of stresses between a matrix and dispersed inclusions. Moreover, the shear stress developed at polyethylene crystals via the sheared EOC matrix overwhelms the plastic resistance of polyethylene crystals at both selected temperatures. These estimations indicate that the deformation of dis-UHMWPE grains into fibrils or nanofibrils is possible by shearing during compounding in an extruder. The necessary conditions are the high viscosity of a polymer matrix and a temperature sufficiently high to decrease the plastic resistance of polyethylene crystals. Indeed, compounding of dis-UHMWPE with viscous EOC in a twin screw extruder leads to strong deformation of crystalline UHMWPE grains into fibrils and nanofibrils.

Sheared polyethylene crystals retain their consistency judging by the high melting temperature after prolonged compounding at 75 °C. At 115 °C some unraveling of polyethylene crystals is seen judging from the crystallinity decrease, albeit with preserved high melting temperature. From the thermal analysis studies it is apparent that melting temperature and melting process strongly depend on the topological constraints present in the amorphous phase of the semi-crystalline UHMWPE. The defects present on the nanofiber surfaces, such as chain folds and amorphous fragments (initial crystallinity was ≈70 wt.%, and 30 wt.% of amorphous fragments are then dispersed along the nanofibers, also decreasing the crystal thickness of a nanofiber) could locally decrease the melting temperature leading to nanofiber local melting and fragmentation. The condition required for the melting of in situ solid-state generated nanofibers of dis-UHMWPE is in good agreement with the prediction of the Gibbs–Thomson equation. The peak at a lower temperature is attributed to the melting of nanofibers, formed at 115 °C, having thinner chain-folded crystals. The crystalline nanofibers with thickened chain-extended crystals persist further with the heating and show a melting peak at higher temperatures, where the melting temperature recorded via DSC goes beyond the equilibrium melting temperature of linear polyethylene due to the transfer of the macroscopic stress to molecular length scale [55]. The elaborated approach not only simplifies the production technology of solid-state in-situ generation of composites, but also deepens our knowledge of the existing limitations on the plastic deformation of nanofibers in the solid state.

## Figures and Tables

**Table 1:** Calorimetric data of dis-UHMWPE powder and EOC/UHMWPE nanocomposites processed at different conditions. *T_m_*, *T_o_*, and *T_e_* denote the melting peak temperature, the onset, and endset melting temperatures, respectively, whereas Δ*H_m_* and *X_c_* are the melting enthalpy, and the degree of crystallinity of dis-UHMWPE calculated based on its melting enthalpy according to Equation (2), respectively.

Sample Code	First Heating					Second Heating				
	*T_o_* [°C]	*T_m_* [°C]	*T_e_* [°C]	Δ*h_m_* [J g^−1^]	*X_c_* [%]	*T_o_* [°C]	*T_m_* [°C]	*T_e_* [°C]	Δ*h_m_* [J g^−1^]	*X_c_* [%]
Dis-UHMWPE	133.5	140.0	144.7	202.0	68	126.8	136.0	140.3.	132.9	45
Comp (75 °C, 10 min)	131.9	138.9	144.4	9.08	62	127.7	134.1	137.9	4.83	33
Comp (75 °C, 30 min)	132.2	138.8	144.2	5.86	40	127.7	134.4	138.0	3.36	23
Comp (75 °C, 90 min)	133.4	139.1	143.8	5.71	39	127.8	134.7	138.1	3.37	23
Comp (115 °C, 10 min)	136.1	144.0 (139.9)	150.5	5.56	38	128.5	135.1	137.6	2.64	18
Comp (115 °C, 30 min)	136.2	143.8 (139.8)	148.8	4.98	34	128.3	135.0	137.8	2.78	19

## Data Availability

The data supporting reported results can be obtained on request from the authors.
